# Supplementary material for: Effectiveness of the Pfizer-BioNTech (BNT162b2) vaccine against the omicron variant of SARS-CoV-2 among adults aged 50 and above: A case-control study in Lebanon, June 2022
Source: PLoS One. 2025 Mar 20;20(3):e0318344. doi: 10.1371/journal.pone.0318344 (PMC11925275; doi:10.1371/journal.pone.0318344)
Supplement: S1 Fig — (PDF) [file pone.0318344.s001.pdf]

S1 Fig.: ***Minimum number of cases and controls to detect for a specified VE, estimated vaccination coverage in the population under evaluation, with 1–3 controls per case, with a precision of ± 10%, and a type 1 error rate of 0.05***

Vaccine effectiveness	Vaccination coverage in the population being studied	1:1 cases to controls		1:2 cases to controls		1:3 cases to controls	
		No. cases	No. controls	No. cases	No. controls	No. cases	No. controls
**50%**	30%	1133	1133	902	1804	825	2475
	50%	828	828	633	1266	568	1704
	70%	855	855	624	1248	546	1638
	90%	1736	1736	1195	2390	1015	3045
**70%**	30%	526	526	441	882	412	1236
	50%	346	346	274	548	250	750
	70%	319	319	234	468	205	615
	90%	580	580	381	762	315	945
**90%**	30%	150	150	138	276	134	402
	50%	80	80	70	140	67	201
	70%	56	56	45	90	41	123
	90%	75	75	48	96	39	117

Source : WHO-2019-nCoV-vaccine-effectiveness-measurement-2021.1-eng.pdf
