# Supplementary material for: Effectiveness of the Pfizer-BioNTech (BNT162b2) vaccine against the omicron variant of SARS-CoV-2 among adults aged 50 and above: A case-control study in Lebanon, June 2022
Source: PLoS One. 2025 Mar 20;20(3):e0318344. doi: 10.1371/journal.pone.0318344 (PMC11925275; doi:10.1371/journal.pone.0318344)
Supplement: S1 Table — (PDF) [file pone.0318344.s002.pdf]

S1 Table. :Estimating Pfizer-BioNTech COVID-19Vaccine

For data management purposes

Investigator name ___________________________	Investigation date ______/_______/________	
Participant record ID	_______________	
Participant identification	1. Case	2. Control
Identification ID (from sampling frame)	__________________	

(Fill from the sampling frame)

First Name	________________
Middle Name (Initials)	_________________
Last Name (Initials)	_________________
Phone number	_________________ ***(if wrong, stop here)***
Sentinel site	__________________
Date of testing	______/_______/_______ (dd/mm/yyyy)

The ministry of health is conducting a study about effectiveness of Pfizer-BioNTech vaccine against COVID-19 disease among Lebanese population > 50 years old and who conducted the PCR test in sentinel sites. You were randomly selected to participate in the study. We hope that you answer the following questions that take 10-15 min, knowing that the data analysis will not include any names. You can also refrain from answering any question or stop answering whenever you wish

Do you Agree to participate in the study?	☐ Yes	☐ No, specify reason for refusal: _________________________

**Part A. General details**	
1. Gender	1. Male 2. Female
2. Date of birth (dd/mm/yyyy)	______/_______/_______ age in years : _______
3. Nationality	1. Lebanese 2.syrian 3. Palestinian 4. Other: _________
4. Locality of residence	__________________
5. District of residence	__________________
6. Province of residence	__________________
7. Clinical symptoms	1.Fever(Y/N) 2.Cough(Y/N) 3.Headache(Y/N)<br>4.Sore Throat(Y/N) 5.Myalgia (Y/N) 6.Diarrhoea (Y/N)<br>7. Vomiting(Y/N) 8.Nausea (Y/N) 9.Malaise(Y/N)<br>10.loss of taste(Y/N) 11.loss of smell(Y/N) 12. Dyspnoea(Y/N)<br>13.Other symptoms (Y/N) specify:____________<br>***(If control with loss of smell or loss of taste, discard from analysis)***
8. Symptoms Onset	_______________(dd/mm/yyyy)
9. Outcome **(for cases only)**	1. Remission (Y/N) 2. Hospitalization (Y/N) 3. Death(Y/N)
9.b Cause of death	1. Post COVID-19 complications (Y/N)<br>2. Other cause(s) (Y/N) specify:_______
10. Smoking status	1. Not a smoker 2. Former smoker 3. Current smoker<br>If smoker, specify number of cigarettes/day: ________<br>If smoker, specify how many times you smoke narguileh /day: _______<br>If smoker, specify how many times you smoke pipe/day: _________
11. Have you been diagnosed with COVID19 3 months before date of testing? If yes, specify date of laboratory confirmation	□ Yes □ No □ Unknown<br>Date of laboratory confirmation : _______________(dd/mm/yyyy)<br>**(*if yes, discard from analysis)***

**Part B. Health status**

1. In the previous 12 months, were you experiencing any of the following medical conditions?				
a) Diabetes	1. Yes, treated with medication	2. Yes, not treated with medication	3. No,	4. Unk
b) Heart disease	1. Yes, treated with medication	2. Yes, not treated with medication	3. No	4. Unk
c) Hypertension	1. Yes, treated with medication	2. Yes, not treated with medication	3. No	4. Unk
d) Immunodeficiency or organ transplant (*congenital or acquired immunodeficiency, and medication with immunosuppressant agents.)*	1. Yes, treated with medication	2. Yes, not treated with medication	3. No	4. Unk
e) Lung disease	1. Yes, treated with medication	2. Yes, not treated with medication	3. No	4. Unk
f) Asthma	1. Yes, treated with medication	2. Yes, not treated with medication	3. No	4. Unk
g) Cancer (Current)	1. Yes, treated with medication	2. Yes, not treated with medication	3. No	4. Unk
h) History of Cancer with remission	1. Yes, treated with medication	2. Yes, not treated with medication	3. No	4. Unk
i) Renal disease	1. Yes, treated with medication	2. Yes, not treated with medication	3. No	4. Unk
j) Liver disease	1. Yes, treated with medication	2. Yes, not treated with medication	3. No	4. Unk
k) Rheumatological disease	1. Yes, treated with medication	2. Yes, not treated with medication	3. No	4. Unk
k) Other : specify:______________________	1. Yes, treated with medication	2. Yes, not treated with medication	3. No	4. Unk
2. In the previous 12 months, did you visit a physician for medical consultation? if yes, specify the number of medical consultation	1. Yes 2. No 3. Unknown<br>Number of medical consultations: _______			
3. In the previous 12 months, were you admitted to hospital? if yes, specify the number of hospital admission	1. Yes 2. No 3. Unknown<br>Number of hospital admissions: _______			

**Part D. Living arrangements**	
1. How many household members live with	______

you (without kitchen and bathroom)?	
2. How many rooms are there in your current housing?	______
3. Are you able to cover your basic needs?	1. I can cover all my basic needs<br>2. I can cover my basic needs with financial difficulties<br>3. I can cover my basic needs partially<br>4. I can't cover my basic needs
4. In the previous 6 months, Prior to your testing date, what was the **main** origin of your personal income?	1. work income<br>2. Retirement payment<br>3. Family's help<br>4.Financial help not family related (i.e. NGO or governmental subsidies)<br>5.Help from abroad<br>6. Personal savings<br>7. No income<br>8. Refuse to answer<br>9. other : __________

**Part E. Exposure to SARS-CoV-2 in the community**				
1. In the previous 14 days preceding COVID-19 testing, did you have a contact with confirmed or suspected COVID-19 case?	1. Yes	2. No		
2. In the previous 6 months preceding COVID-19 testing, did you use public transportation?	1. Always	2.Sometimes	3. Never	4. Not applicable
3. In the previous 6 months preceding COVID-19 testing, did you attend social events and gatherings?	1. Always	2.Sometimes	3. Never	4. Not applicable
4. In the previous 6 months preceding COVID-19 testing, did you travel?	1. Always	2.Sometimes	3. Never	4. Not applicable
**Part F. practice of non-pharmaceutical interventions (NPI)**				

1. In the previous 6 months preceding COVID-19 testing, did you use mask when going outdoor?	1. Always 2.Sometimes 3. Never 4. Not applicable
2. In the previous 6 months preceding COVID-19 testing, did you ensure social distancing in public?	1. Always 2.Sometimes 3. Never 4. Not applicable
3. In the previous 6 months preceding COVID-19 testing, how often did you wash your hands with soap and water before touching your face?	1. Always 2.Sometimes 3. Never 4. Not applicable
4. In the previous 6 months preceding COVID-19 testing, how often did you use hand sanitizer when soap and water are not available	1. Always 2.Sometimes 3. Never 4. Not applicable

**Part G: Vaccination details**	
1. Did you receive a vaccine against COVID-19before testing?	1. Yes 2. No 3.Unknown
a. If no, specify the reason	1. I have contraindication for COVID19 vaccination (Y/N) ***(if yes, discard from analysis)***<br>2. Got infected before my turn (Y/N)<br>3. Vaccination centre inaccessible (Y/N)<br>4. Fear of side effects (Y/N)<br>5. Doubt of its effectiveness (Y/N)<br>6. Not aware of vaccination (Y/N)<br>7.Fear of contacting COVID-19 (Y/N)<br>Other reasons, specify : ________________
If vaccinated,	
4. Did you receive Pfizer-BioNTech vaccine before testing date?	1. Yes 2. No 3.Unknown<br>***If other than Pfizer vaccine or***

	unknown, discard from analysis
5. If yes, how many doses	□ 1 □ 2 □ Unknown
6. What was the date of the first dose?	______/_______/_______ (dd/mm/yyyy)<br>***If the date of the testing less than 3 weeks following vaccination, discard from analysis***
7. What was the phone number you used for registration ?	_________________________

8. Did you experience any effect in the first 3 days after your first vaccine dose?	1. No side effects<br>2. Minor side effects (pain at injection site, headache, fatigue, fever, chills, myalgia,…)<br>3. Moderate side effects limiting daily activities (asthenia, tachycardia, …)<br>4. Severe side effects requiring hospitalization<br>5. Death, if yes, specify date: ______/_______/_______
9. What was the date of the second dose? (dd/mm/yyyy)	______/_______/_______ (dd/mm/yyyy)
10. Did you experience any effect in the first 3 days after your second vaccine dose?	1. No side effects<br>2. Minor side effects (pain at injection site, headache, fatigue, fever, chills, myalgia,…)<br>3. Moderate side effects limiting daily activities (asthenia, tachycardia, …)<br>4. Severe side effects requiring hospitalization<br>5. Death, if yes, specify date: ______/_______/_______
