# Supplementary material for: Effectiveness of the Pfizer-BioNTech (BNT162b2) vaccine against the omicron variant of SARS-CoV-2 among adults aged 50 and above: A case-control study in Lebanon, June 2022
Source: PLoS One. 2025 Mar 20;20(3):e0318344. doi: 10.1371/journal.pone.0318344 (PMC11925275; doi:10.1371/journal.pone.0318344)
Supplement: Attachment — Submitted filename: Response to Reviewers.docx [file pone.0318344.s004.docx]

We sincerely appreciate the reviewers' thoughtful feedback and have made revisions accordingly to enhance the clarity and depth of our manuscript. Below is a detailed table that outlines each of the reviewers' comments, along with our corresponding responses and the actions taken to address them.

| Response to Reviewer One: Thank you for your constructive feedback, which helped improve the clarity and quality of our manuscript. | |
| --- | --- |
| Comment 1: In the abstract, there are redundant ideas present in these two paragraphs. It is preferable to consolidate this idea into a single, clear statement to avoid duplication.  “Factors such as age, province of residence, and underlying conditions significantly influenced VE. The study underscores the need for on-going monitoring and potential booster doses to enhance protection, especially against evolving variants like Omicron.”  “Factors such as age, province of residence, and underlying conditions exert significant influence on VE, highlighting the need for tailored vaccination strategies. Booster doses may play a crucial role in enhancing VE and mitigating the impact of evolving strains like Omicron.” | We agree that the two paragraphs in the abstract contained redundant ideas. We have revised and consolidated them into a single, clearer statement as follows:  Factors such as age, province of residence, and underlying conditions significantly influenced VE. The study underscores the need for an on-going monitoring and potential booster doses to enhance protection, especially against evolving variants like Omicron and emphasizes the importance of proactive measures in safeguarding public health amidst evolving viral threats. |
| Comment 2: There seems to be an error in the percentage calculations in the following paragraph in the introduction section. Please review and update the percentages accordingly.  “Whereas in Lebanon as of 17 February 2022 5,779,829 vaccine administered in total (7). The Pfizer, BioNTech vaccine was the most administered with 4,253,218 doses (82,9%) followed by AstraZeneca 717,304 (13.9%); added together they make 96.8 % of the brands administered.” | We have reviewed the numbers and corrected the percentages accordingly. The revised paragraph now reads:  Whereas in Lebanon as of 17 February 2022 5,779,829 vaccine had been administered in total by the same time[7] .The Pfizer, BioNTech vaccine was the most administered with 4,253,218 doses (73.58 %) followed by AstraZeneca 717,304 (12.41%); added together they make 85.9% of the total brands administered. |
| Comment 3: “Introduction: This study aims to highlight the effectiveness of the Pfizer-BioNTech vaccine brand against the Omicron variant of SARS-CoV-2, focusing on individuals aged 50 and above presenting with influenza-like illness at sentinel sites.”  Could you please explain why you chose to study this specific population? If there is a particular reason, it would be more informative to include this rationale in the methodology section of the article. | We chose to focus on individuals aged 50 and above in this study because in Lebanon, the Pfizer-BioNTech vaccine was primarily administered to healthcare workers and individuals in this age group due to their higher risk of severe outcomes from COVID-19. Clarification was added in the methodology section. |
| Comment 4: Please include relevant dates in the methodology section, specifically the periods of patient recruitment. | We have updated the methodology section to include the relevant dates for patient recruitment. The revised sentence now reads:  Data collection was conducted from February till the end of April. |
| Comment 5: The methodology is clear and well written. However, I recommend creating a specific section for data analysis instead of merging it with study design or data collection sections. For example:  “The outcome of interest for the primary analysis will be the detection of SARS-CoV-2 in patients eligible for vaccination and presented to ILI/CLI sites with symptoms fitting the case definition.” This information was stated in the study design section. However, this should be mentioned in the data analysis section.  “Data analysis was done using R version 4.0.2 and R Studio. The measure of association is an odds ratio (OR). This can be measured by logistic regression taking into considerations all the confounding factors cited in the literature.” This information was stated in the data collection section. However, this should be mentioned in the data analysis section.  In addition, it would be beneficial to provide more details on how quantitative variables were handled in the analyses and describe all statistical methods used. | A new section was added as suggested :  Data analysis:  The outcome of interest for the primary analysis was the detection of SARS-CoV-2 in patients eligible for vaccination and presented to ILI/CLI sites with symptoms fitting the case definition. Data analysis was done using R version 4.0.2 and R Studio. Descriptive statistics were calculated to summarize the demographic and clinical characteristics of the participants, frequencies and numbers were reported for categorical variables and comparisons of percentages between groups were made using Pearson's Chi-square tests for categorical variables to assess the significance of observed differences. The measure of association was the odds ratio (OR). This was can be measured by the logistic regression taking into considerations all the confounding factors cited in the literature. |
| Comment 6: How were the confounders chosen? Did you base your selection solely on the literature, or did you also consider the results of the bivariate analysis? | Explanation was added to the methods section:  The selection of confounders was based on the guideline by WHO for VE studies. Following this, we performed bivariate analyses to explore associations between these factors and the outcome of interest and all variables with p value less than 0.2 were added. This dual approach allowed us to ensure that our confounder selection was comprehensive and grounded in both theoretical and empirical evidence. The chosen confounders included age groups, gender, province of residence, smoking status, underlying conditions, coverage of basic needs, recent contact with a COVID-19 case, exposure to public transportation, social events, travel, mask use, social distancing, handwashing, hand sanitizer use, and nationality. |
| Comment 7: The result section is well-structured. However, there is a replication of results between the text and the tables; for example, the disease manifestations paragraph repeats all the results of table 2. To avoid duplication, it is recommended to report only the most prevalent manifestations or the significant results in the text. The remaining variables can be presented in the table. | To enhance clarity and reduce redundancy, we revised the results section and adjusted accordingly. |
| Comment 8: In the result section, specifically the “Table 2: Logistic regression adjusted to confounders with OR”, it is recommended to add the confidence interval and p-value of the OR.  In addition, in this same table, it would be beneficial to add the results of the confounding variables considered in the logistic regression model, and not to present only the vaccination status variable. (you can present at least the variables with significant results). | We have revised “Table 2” and adjusted the title to “table 8”: Logistic regression adjusted to confounders with OR” to include the confidence intervals (CIs) and p-values for the odds ratios (OR), as requested. Additionally, we have expanded the table to include the results for the confounding variables considered in the logistic regression model. |
| Comment 9: In the discussion section, you mentioned that: “An OR less than one were seen for the fever, headache, nausea, myalgia, vomiting, loss of taste, loss of smell this is seen when the vaccination plays a protective effect against one of the symptoms.” However, according to the table 3, the P-value for “headache” is 0.212, which is a not statistically significant. This needs to be adjusted in the text. | Adjusted upon your comment to be: However, while the OR for headache and dyspnea was also less than one, this result was not statistically significant. |
| Comment 10: The conclusion section is well-written, summarizing the main results and outlining the implications for future research. However, I suggested moving the first paragraph of the conclusion (“Knowing that we have limited facts --> for the Gamma variant during the same time frame (17)”) to the discussion section. This paragraph compares the study results with other studies conducted in the UK and South Africa, making it more appropriate for the discussion section. | We agree that the first paragraph of the conclusion, which compares the study results with other studies conducted in the UK and South Africa, is more suited to the discussion section. We have now moved the paragraph to the discussion section. |
| Comment 11: Please review the numbering of the tables. | Reviewed and adjusted. |
| Response to Reviewer Two: thank you for your detailed review and insightful suggestions. Your input has significantly contributed to refining our work. | |
| Comment 1: Please ensure to state what acronym stand for when the acronym is used for the first time (eg. SARI in study design instead of study population). | It was adjusted accordingly and we have also reviewed the document to confirm that other acronyms are similarly defined upon their first use to enhance clarity for readers. |
| Comment 2: Also please keep consistent towards the terminologies used and avoid typos (SARS-CoV-2 vs SARSCoV-2 in study design section). | We have carefully reviewed the manuscript to ensure that the terminology is consistent throughout the document. |
| Comment 3: Ethical consideration section should have been in past tense (why were “will” and “should” included in this part?) | We have revised the section accordingly. |
| Comment 4: The manuscript needs to go through an overhaul in terms of organization. It is unclear what the table containing the chronic diseases right underneath table 1 is? Also, table formats should be consistent. | Thank you for your feedback. We recognize the importance of a clear and consistent structure throughout the manuscript. The table containing chronic diseases beneath Table 1 was unintentionally misplaced and was relocated to the appropriate section for clarity. Additionally, we ensured that all table formats are consistent. |
| Comment 5: Please keep spacing and spacing before/after punctuation consistent. | Thank you for highlighting this. We have thoroughly reviewed the entire manuscript to ensure consistency in spacing. |
| Comment 6: The manuscript should be read very carefully to correct typos and grammatical issues. Many typos and punctuation errors exist. | The manuscript has undergone a comprehensive review to address and correct all typos, grammatical issues, and punctuation errors. |
| Comment 7: A more elaborate discussion / conclusion is required to ensure that results are thoroughly discussed and elaborated on. | We have expanded both the discussion and conclusion sections to provide a more comprehensive analysis of our results. |
| Comment 8: The authors should check to see if there is any correlation between the timing of vaccination and the symptoms observed. For instance, previous work (Barin et al., Lancet Microbe, 2022 and others) demonstrated a significant reduction in antibody titers over time so it is crucial to observe if the symptom severity is reduced in recently vaccinated individuals. Please carry out correlation studies in the light of different vaccination times and symptoms experienced. | Thank you for your valuable suggestion regarding the correlation between the timing of vaccination and the symptoms observed.  However, due to the constraints of our current dataset, we were unable to perform a thorough correlation analysis between vaccination timing and the symptoms experienced by participants. We emphasized this limitation in the revised manuscript and suggest it as a direction for future studies. |
| Comment 9: Why weren’t the results for thyroid disorders, neurological disorders and autoimmune diseases included in the report? This could provide interesting and novel information. | The primary reason these results were not included in the report is due to limitations in our sample size and the statistical power needed to analyze these specific disorders. During the analysis, we found that the prevalence of these conditions within our study population was low and were cited as others in free text, which limited the ability to draw meaningful conclusions or to perform robust statistical analyses. |
