# Supplementary material for: Effectiveness of the Pfizer-BioNTech (BNT162b2) vaccine against the omicron variant of SARS-CoV-2 among adults aged 50 and above: A case-control study in Lebanon, June 2022
Source: PLoS One. 2025 Mar 20;20(3):e0318344. doi: 10.1371/journal.pone.0318344 (PMC11925275; doi:10.1371/journal.pone.0318344)
Supplement: Attachment — Submitted filename: Response to Reviewers 1.docx [file pone.0318344.s005.docx]

Greetings,

Response to Reviewers:

Thank you for your valuable feedback which has helped enhancing the clarity and precision of our study and for acknowledging that the previous comments have been addressed sufficiently.

**Review comment to the Author:**

Reviewer #3: My previous comments have been sufficiently addressed. Some things to add would be to state which version of Pfizer Biontech vaccine was implemented and considered during study time as many different updated vaccines emerged. Some of those were more effective against omicron than others. Similarly, please clarify what is meant by “fully vaccinated” in the “Definition of vaccination status:” section highlighting which vaccine version was considered. The originally implemented ones? If so please clarify accordingly.

I agree with the statement in the discussion which states: Key findings of the study indicate that the Pfizer-BioNTech vaccine is effective in reducing the odds of developing symptomatic COVID-19. This should have been highlighted more and emphasized in the abstract as it is an important finding. The abstract reads almost as vaccination not having an enough positive impact where the authors' key findings clearly indicates otherwise. Please update accordingly.

Response to reviewer #3:

**Response to Reviewer #3**

. Regarding your additional suggestions:

1. **Pfizer-BioNTech Vaccine Version**:
   We appreciate your suggestion to specify the version of the Pfizer-BioNTech vaccine implemented during the study. During the study period, the original Pfizer-BioNTech COVID-19 vaccine (BNT162b2) was the version available and administered in Lebanon. We have updated the manuscript to clarify this point.
2. **Definition of “Fully Vaccinated”**:
   We have clarified the definition of “fully vaccinated” in the “Definition of vaccination status” section. In the context of this study, “fully vaccinated” refers to individuals who received the complete initial series of the original Pfizer-BioNTech COVID-19 vaccine (two doses of BNT162b2). This definition has been adjusted in the manuscript to avoid any ambiguity.
3. **Abstract adjustments**:

**W**e have revised the abstract to state the protective effects of vaccination, including its role in mitigating symptom severity.

Thank you again for your thoughtful remarks.

Dear editors,

Thank you for your feedback. We have reviewed the reference list and made the necessary updates and adjustments as per your recommendations. The following changes have been made:

1. **Reference 2**: The link to the Centers for Disease Control and Prevention (CDC) website has been updated as follows:
   - Centers for Disease Control and Prevention. (n.d.). Ncov 2019 – Variants classifications. Available from: <https://www.cdc.gov/covid/?CDC_AAref_Val=https://www.cdc.gov/coronavirus/2019-ncov/variants/variant-classifications.html> (visited 20-9-2022).
2. **References 5 and 13**: Typographical errors were corrected as follows:
   - Tan, S. H., Cook, A. R., Heng, D., Ong, B., Lye, D. C., Tan, K. B., et al. (2022). Effectiveness of BNT162b2 vaccine against Omicron in children 5 to 11 years of age. *New England Journal of Medicine, 387*(6), 525–532. <https://doi.org/10.1056/NEJMoa2203209>.
   - Li, Q. H., Ma, Y. H., Wang, N., Hu, Y., & Liu, Z. Z. (2020). New coronavirus-infected pneumonia Engulfs Wuhan. *Asian Toxicology Research, 1*, 1–7.

Best Regards,
